# Gibberellic Acid and Jasmonic Acid Improve Salt Tolerance in Summer Squash by Modulating Some Physiological Parameters Symptomatic for Oxidative Stress and Mineral Nutrition

**DOI:** 10.3390/plants10122768

**Published:** 2021-12-15

**Authors:** Mashael M. Al-harthi, Sameera O. Bafeel, Manal El-Zohri

**Affiliations:** 1 Department of Biology, Faculty of Applied Science, Umm Al-Qura University, Makkah 21955, Saudi Arabia; mmharthy@uqu.edu.sa; 2 Department of Biological Sciences, Faculty of Science, King Abdulaziz University, Jeddah 21488, Saudi Arabia; sbafil@kau.edu.sa; 3 Department of Botany and Microbiology, Faculty of Science, Assiut University, Assiut 71516, Egypt

**Keywords:** phytohormones, salinity, *Cucurbita pepo* (L.), mineral uptake, proline, lipid peroxidation, antioxidant enzymes, nucleic acids

## Abstract

Gibberellic acid (GA) and jasmonic acid (JA) are considered to be endogenous regulators that play a vital role in regulating plant responses to stress conditions. This study investigated the ameliorative role of GA, JA, and the GA + JA mixture in mitigating the detrimental effect of salinity on the summer squash plant. In order to explore the physiological mechanisms of salt stress alleviation carried out by exogenous GA and JA, seed priming with 1.5 mM GA, 0.005 mM JA, and their mixture was performed; then the germinated summer squash seedlings were exposed to 50 mM NaCl. The results showed that a 50 mM NaCl treatment significantly reduced shoot and root fresh and dry weight, water content (%), the concentration of carotenoid (Car), nucleic acids, K^+^, and Mg^++^, the K^+^/Na^+^ ratio, and the activity of catalase (CAT) and ascorbate peroxidase (APX), while it increased the concentration of proline, thiobarbituric acid reactive substances (TBARS), Na^+^, and Cl^−^ in summer squash plants, when compared with the control. However, seed priming with GA, JA and the GA + JA mixture significantly improved summer squash salt tolerance by reducing the concentration of Na^+^ and Cl^−^, TBARS, and the Chl a/b ratio and by increasing the activity of superoxide dismutase, CAT, and APX, the quantities of K^+^ and Mg^++^, the K^+^/Na^+^ ratio, and the quantities of RNA, DNA, chlorophyll b, and Car, which, in turn, ameliorated the growth of salinized plants. These findings suggest that GA and JA are able to efficiently defend summer squash plants from salinity destruction by adjusting nutrient uptake and increasing the activity of antioxidant enzymes in order to decrease reactive oxygen species accumulation due to salinity stress; these findings offer a practical intervention for summer squash cultivation in salt-affected soils. Synergistic effects of the GA and JA combination were not clearly observed, and JA alleviated most of the studied traits associated with salinity stress induced in summer squash more efficiently than GA or the GA + JA mixture.

## 1. Introduction

Salinity stress results in extensive crop damage worldwide; most field crops are salt sensitive, and the problem is expected to increase in the coming decades [1]. Salinity is regarded as the most limiting and damaging of the factors that restrict crop growth, yield, and productivity [2]. Salt-affected soils negatively affect plants in various ways including through water stress, ion toxicity, nutritional imbalance, oxidative stress owing to the formation of reactive oxygen species, alterations to metabolic processes, reductions in photosynthesis rate, membrane damage, declines in cell division and expansion, and genetic disorders [3,4]. Plants have evolved many mechanisms to alleviate the adverse effects of salt stress, including plasticity in changing their morphological patterns, the accumulation of compatible solutes to preserve cell water content and prevent ultrastructural destruction, ion-homeostasis, enhanced water-use efficiency, improved photosynthesis activity, the detoxification of ROS via the activation of antioxidant systems, and the stimulation of plant hormones [5].

It is fundamentally important to recognize how plants perceive stress signals and respond to various environmental stress factors [6]. Plant hormones are active members of the signal cascade involved in the generation of plant responses to stress conditions [7]. The exogenous application of biological growth-promoting substances is a promising sustainable strategy to encourage plant growth and yield and to reinforce the plant’s capacity to alleviate stress conditions [8]. Phytohormones are endogenously produced organic substances essential for regulating plant growth and productivity. Many phytohormones including, abscisic acid (ABA), gibberellins, ethylene, salicylic acid (SA), and jasmonic acid (JA) seem to be critical modules of complex signaling networks and have been integrated into current models of stress response [9]. Hence, they play an important role in prompting plant tolerance to various stress conditions.

Numerous works have proven the potential of gibberellic acid (GA) as a classical plant hormone. Gibberellins (GAs) are phytohormones that regulate numerous metabolic pathways, the activity of many enzymes, and gene expression; therefore, they play a vital role in seed germination and seedling growth, stem and root elongation, leaf expansion, and flowering [10]. The exogenous application of GA3 enhances stomatal conductance, water-use efficiency, photosynthesis activity, ion uptake, and the balance of other phytohormones [11]. Furthermore, GA3 maximizes the antioxidant capacity and osmoprotectants, while it minimizes lipid peroxidation, in order to alleviate the drastic effects of environmental stress [12]. Jasmonic acid, which is affiliated with the new phytohormone group, is a key regulator that plays a major role in plant growth and development and in the mitigation of both biotic and abiotic stress conditions [13]. Understanding of the complexity of the signaling network in which JA is involved is just developing [14]. Jasmonates exert their effects by orchestrating large-scale changes in gene expression [15,16].

Some investigations have shown that there is a relation between GAs and JAs under both usual and adverse conditions. Achard et al. [17], find that GA signaling declines under cold stress, causing plant growth reduction, while DELLA proteins accumulate. Wingler et al. [18] concluded that DELLA proteins can bind to JAZ proteins, causing the stimulation of jasmonate-responsive genes. This outcome reveals JA–GA crosstalk under environmental stress. Like other plant hormones, they exhibit stimulating and inhibiting activities in planta, and synergistic or antagonistic effects with respect to other plant hormones are well known [19].

Summer squash (*Cucurbita pepo* L.) is a popular, widely used crop worldwide, which belongs to family Cucurbitaceae. It grows under tropical and subtropical conditions during summer [20]. It is significant not only because of its utilization as human food but also because of its use as a medicinal plant due to its high content of zinc and antioxidants [21]. Summer squash is a sensitive crop for abiotic stress conditions including salinity [22]. Therefore, it has been chosen as a case study plant in this investigation of how tolerance to salinity stress is improved by seed priming with GA and JA. Several studies have shown that exogenous GA can mitigate toxic effects and improve the vegetative and reproductive responses of many plants under salinity stress [10,11,12]. Nevertheless, there is not a lot of data regarding summer squash and JA.

It is convenient to describe the role played by each phytohormone in salinity tolerance, as if each one worked separately; however, it is well known that phytohormone responses include extensive crosstalk and regulatory interfaces. In some cases, enhancing the signal of several phytohormones is needed for the activation of one stress tolerance gene. In other cases, the induction of one hormone repudiates the action of another. Still another form of crosstalk occurs when one hormone enhances or suppresses the biosynthesis or action of another. Unravelling these interfaces is one of the most urgent issues in plant stress physiology. Therefore, this present study was conducted to explore the interactive responses of salinity stressed summer squash plants to seed priming with GA and JA, as well as the GA + JA mixture, in order to evaluate whether the combination of GA and JA has synergistic effects in alleviating salinity stress in summer squash.

## 2. Results

### 2.1. Biomass and Water Content Percentage

**Figure 1:**
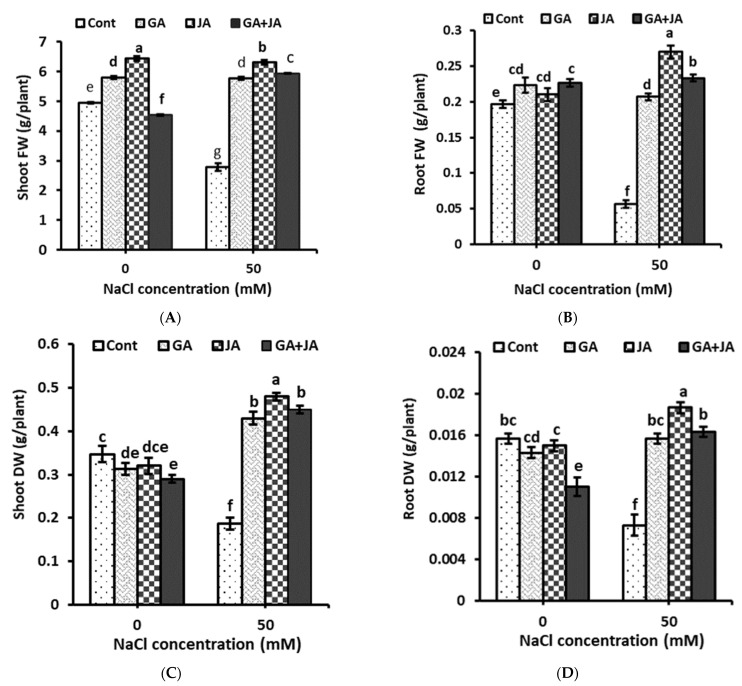
Biomass—(**A**) shoot fresh weight (FW); (**B**) root FW; (**C**) shoot dry weight (DW); and (**D**) root DW—of non-salinized and salinized summer squash plants, as affected by seed priming with 1.5 mM GA, 0.005 mM JA, and mixture of them. Data represent mean of three replicates (*n* = 3) with error bars indicating standard error of the mean. Bars carrying different lowercase letters are significantly different at *p* < 0.05 according to Duncan’s multiple range test. *p* values for two-way ANOVA are reported in Table 1.

The shoot and root biomass of summer squash plants significantly reduced in response to saline conditions. All investigated hormonal treatments significantly enhanced shoot and root fresh and dry weight under saline conditions. The highest values of shoot and root fresh and dry weight under saline conditions were observed for JA treatment (Figure 1). Seed priming with JA caused a significant enhancement in summer squash shoot and root fresh weights under saline conditions, by about 126% and 376%, respectively, as compared to their corresponding controls (Figure 1A,B). GA and the GA + JA mixture enhanced the shoot fresh weight to comparable values of about 107% higher than the saline control (Figure 1A). Under non-saline conditions, GA and JA significantly enhanced shoot fresh weights by about 17% and 30%, respectively, as compared to their corresponding controls (Figure 1A). Under saline conditions, JA caused a significant enhancement in summer squash shoot and root dry weights of about 157% and 155%, respectively, as compared to their corresponding controls. GA and the GA + JA mixture enhanced dry weight under saline conditions in shoots by about 130% and 141% as compared to the control, and in roots by about 114% and 123% as compared to the control (Figure 1C,D). The two-way ANOVA indicated highly significant (*p* < 0.001) effects of both salinity and the salinity × hormones interaction on shoot and root FW and DW. Hormonal treatments showed highly significant (*p* < 0.01) and significant (*p* < 0.05) effects on shoot and root FW, respectively, but no significant effects were shown for individual hormonal treatments on shoot and root DW (Table 1).

**Figure 2:**
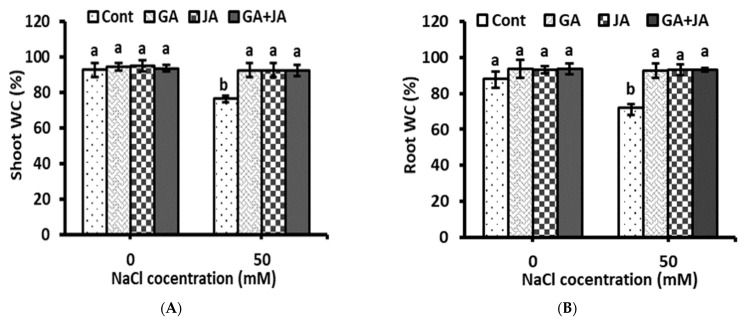
Percentage of water content (WC) in (**A**) shoot and (**B**) root of non-salinized and salinized summer squash plants, as affected by seed priming with 1.5 mM GA, 0.005 mM JA, and mixture of them. Data represent mean of three replicates (*n* = 3) with error bars indicating standard error of the mean. Bars carrying different lowercase letters are significantly different at *p* < 0.05 according to Duncan’s multiple range test. *p* values for two-way ANOVA are reported in Table 1.

The shoot and root water content (WC) percentage was reduced significantly with salinity stress (Figure 2). None of the hormonal treatments, when applied to non-salinized plants, showed any significant effect on WC. However, when salinized plants were treated with all hormonal treatments, their shoot and root WC increased by 20% and 30%, respectively, higher than stressed untreated plants. The two-way ANOVA showed significant effects (*p* < 0.05) of salinity stress and highly significant effects (*p* < 0.01) of the salinity × hormones interaction on shoot and root WC, while no significant effects were shown for individual hormonal treatments (Table 1).

### 2.2. Plant Pigments

Salinity stress did not affect Chlorophyll a (Chl a), while it significantly enhanced Chlorophyll b (Chl b) and reduced the Chl a/b ratio and Carotenoid (Car) concentrations. All hormonal treatments significantly inhibited the Chl a concentration, so that it was lower than that of the controls both under saline and non-saline conditions. The Chl a content was about 34%, 21%, and 46% lower than that of the control after treatment with GA, JA, and GA + JA mixture, respectively, under saline conditions. On the other hand, all hormonal treatments significantly increased the Chl b concentration, as compared to controls under both non-saline and saline conditions. In the best results recorded for JA treatment, it enhanced Chl b concentration by about 5-fold and 2-fold under non-saline and saline conditions, respectively, as compared with their corresponding controls. Seed priming with GA and the GA + JA mixture enhanced Chl b accumulation at comparable rates under non-saline and saline conditions. In the same context, all hormonal treatments significantly decreased the Chl a/b ratio, under non-saline and saline conditions, to lower levels than their corresponding controls. GA, JA, and the GA + JA mixture inhibited the Chl a/b ratio, under saline conditions, by about 59%, 63%, and 63% and, under non-saline conditions, by about 77.5%, 81%, and 77%, compared to their respective controls. Carotenoid concentration was significantly enhanced by hormonal treatments under saline and non-saline conditions. The highest values of Car were recorded for plants treated with JA; they were 24% and 58% higher than the controls under non-saline and saline conditions respectively. GA and the GA + JA mixture enhanced Car accumulation under saline conditions by about 18% and 14% and, under non-saline conditions, by about 22% and 31%, as compared to the respective controls (Table 2). The two-way ANOVA showed highly significant effects of salinity stress on Chl b concentration (*p* < 0.001) and Chl a/b ratio (*p* < 0.01) and a significant effect on Car concentration (*p* < 0.05), while no significant effect was observed for salinity stress on Chl a concentration. All hormonal treatments and the salinity × hormones interaction showed highly significant effects (*p* < 0.001) on Chl a, Chl b, and Car concentration and the Chl a/b ratio (Table 2).

### 2.3. Mineral Ions

The plants’ mineral uptake was strongly influenced by both salinity and hormonal treatments (Table 3). Na^+^ concertation significantly increased in plant shoots and roots in response to saline conditions, while all hormonal treatments significantly reduced Na^+^ concentration in plant shoots and roots to lower levels than those of the corresponding stressed controls. Under saline conditions, GA, JA, and the GA + JA mixture reduced Na^+^ concentration in shoots by about 21%, 61%, and 58%, and in roots by about 56%, 39%, and 54%, compared to their respective controls. Salinity stress significantly reduced K^+^ concentration and the K^+^/Na^+^ ratio in plant shoots and roots. Under saline conditions, only GA treatment enhanced Na^+^ concentration to a significantly higher level than that of the stressed control in shoot samples. In plant roots, seed priming with GA, JA, and the GA + JA mixture significantly enhanced K^+^ concentration by about 36.1%, 89.6%, and 59.74%, as compared to the stressed control. Salinity stress significantly reduced the K^+^/Na^+^ ratio in plant roots and shoots. All hormones significantly enhanced the K^+^/Na^+^ ratio under saline conditions to higher levels than in stressed controls. GA, JA, and the GA + JA mixture increased the K^+^/Na^+^ ratio in summer squash shoots by about 47.15%, 122.76%, and 156.09%, respectively, as compared to the saline control. The alleviative effect of seed priming with the studied treatments on the K^+^/Na^+^ ratio was more pronounced in the root samples. GA, JA, and the GA + JA mixture increased the K^+^/Na^+^ ratio in plant roots by about 3-fold, as compared to the stressed control (Table 3).

Salinity stress noticeably reduced Mg^++^ concentration in both plant shoot and root. Under saline conditions, seed priming with JA and the GA + JA mixture significantly enhanced Mg+ concentration in summer squash shoots and roots to higher levels than those of the stressed controls. JA and the GA + JA mixture enhanced Mg^++^ concentration in shoots under saline conditions by about 14.41% and 12.40%, as compared to the stressed control. In root samples, seed priming with JA and the GA + JA mixture enhanced Mg^++^ concentration in shoots under saline conditions by about 80.24% and 55.45%, respectively, compared to the stressed control (Table 3).

The concentration of chloride ions markedly increased in both shoot and root of summer squash in response to salt treatment. Seed priming with GA significantly reduced Cl^−^ concentration of summer squash shoot under saline conditions by about 17.6%, as compared to the stressed control. All hormonal treatments significantly reduced Cl^−^ concentration of summer squash roots under saline conditions. GA, JA, and the GA + JA mixture decreased Cl^−^ concentration under saline conditions in roots by about 21.04%, 26.11%d and 13.35%, respectively, as compared to the stressed control. Under non-saline conditions, seed priming with JA significantly reduced Cl^−^ concentration in the shoot by about 31.33% and enhanced it in the root by about 21.22%, as compared to their corresponding unstressed controls (Table 3). The two-way ANOVA showed highly significant (*p* < 0.001; *p* < 0.01) effects of salinity stress on the concentration of mineral ions both in shoots and roots; hormonal treatments showed highly significant (*p* < 0.01) effects on Na^+^ and K^+^ concentrations and the Na^+^/K^+^ ratio in plant shoots and roots, and on the Cl^−^ concentration in roots, while no significant effects were observed for hormonal treatments and the salinity × hormones interaction on Mg^++^ and Cl^−^ in the plant shoots. The salinity × hormones interaction showed highly significant effects (*p* < 0.001) on Na^+^ and the Na^+^/K^+^ ratio in plant shoots and on all studied mineral ions in roots, as well as significant effects (*p* < 0.05) on K^+^ in shoots (Table 3).

### 2.4. Proline

**Figure 3:**
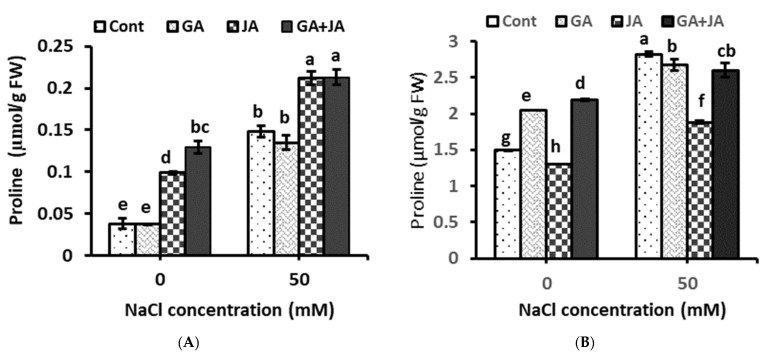
Proline concentration in (**A**) shoot and (**B**) root of non-salinized and salinized summer squash plant as affected by seed priming with 1.5 mM GA, 0.005 mM JA, and mixture of them. Data represent mean of three replicates (*n* = 3) with error bars indicating standard error of the mean. Bars carrying different lowercase letters are significantly different at *p* < 0.05 according to Duncan’s multiple range test. *p* values for two-way ANOVA are reported in Table 1.

The proline content significantly increased in plant shoots and roots due to salinity stress. Seed priming with JA and the GA + JA mixture significantly enhanced proline accumulation in the shoot system under saline and non-saline conditions to higher levels than in their corresponding controls (Figure 3A). JA and the GA + JA mixture showed significant increase in proline concentration under saline conditions in shoots, by about 42% and 43%, respectively, as compared to the stressed control. Under non-saline conditions, JA and the GA + JA mixture significantly enhanced the proline concentration in shoots by about 160% and 240%, respectively, as compared to the control (Figure 3A). In roots, GA and the GA + JA mixture significantly enhanced proline concentration under non-saline conditions by about 37% and 46%, respectively, as compared to the control. Seed priming with all investigated hormonal treatments did not enhance proline accumulation in plant roots under salt stress (Figure 3B). The two-way ANOVA indicated highly significant effects of salinity stress (*p* < 0.001) and hormonal treatments (*p* < 0.01) on proline concentration in plant shoots and roots. The interactive effect of the salinity × hormones interaction was highly significant (*p* < 0.01) on proline concentration in plant shoots and significant (*p* < 0.05) on proline concentration in roots (Table 1).

### 2.5. Thiobarbituric Acid Reactive Substances (TBARS)

**Figure 4:**
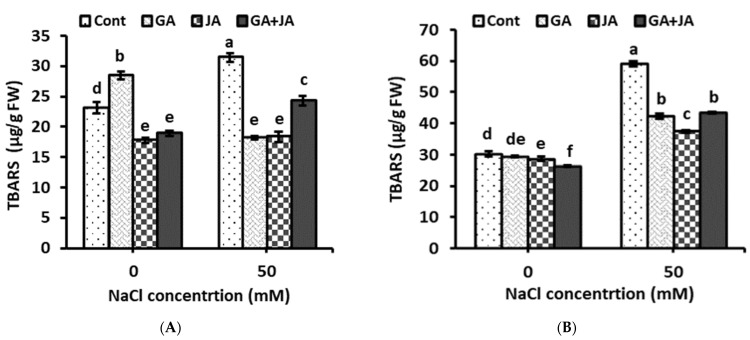
Concentrations of thiobarbituric acid reactive substances (TBARS) in (**A**) shoot and (**B**) root of non-salinized and salinized summer squash plants, as affected by seed priming with 1.5 mM GA, 0.005 mM JA, and mixture of them. Data represent mean of three replicates (*n* = 3) with error bars indicating standard error of the mean. Bars carrying different lowercase letters are significantly different at *p* < 0.05 according to Duncan’s multiple range test. *p* values for two-way ANOVA are reported in Table 1.

The accumulation of lipid peroxidation products is the main sign of ROS acting on the bio-membrane. As shown in Figure 4, all hormonal treatments alleviated stress-induced lipid peroxidation, in terms of TBARS content, both in plant shoots and roots. Under saline conditions, GA, JA, and the GA + JA mixture decreased TBARS concentration in shoots by about 42%, 41%, and 22%, and in root by about 28%, 36%, and 26%, as compared to their corresponding controls. The two-way ANOVA indicated highly significant effects of salinity stress (*p* < 0.001) and significant effects of hormonal treatments and their interaction (*p* < 0.05) on TBARS content in plant shoots. Salinity stress, hormonal treatments, and their interaction showed highly significant effects (*p* < 0.001) on TBARS concentration in plant roots (Table 1).

### 2.6. Antioxidant Enzymes

**Figure 5:**
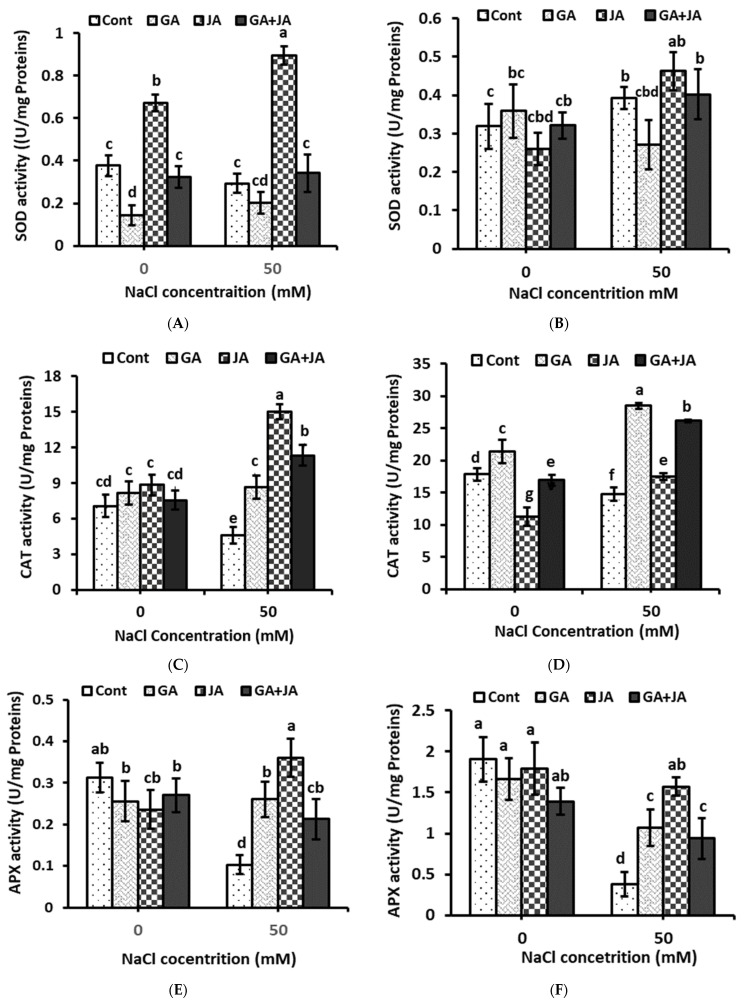
Antioxidant enzyme activity—(**A**) Superoxide dismutase (SOD) in shoot; (**B**) SOD in root; (**C**) Catalase (CAT) in shoot; (**D**) CAT in root; (**E**) Ascorbate peroxidase (APX) in shoot; and (**F**) APX in root—of non-salinized and salinized summer squash plants, as affected by seed priming with 1.5 mM GA, 0.005 mM JA, and mixture of them. Data represent mean of three replicates (*n* = 3) with error bars indicating standard error of the mean. Bars carrying different lowercase letters are significantly different at *p* < 0.05 according to Duncan’s multiple range Test. *p* values for two-way ANOVA are reported in Table 1.

The data represented in Figure 5 revealed that salt treatment significantly reduced CAT and APX activity in summer squash shoots and roots compared to unstressed controls. Only JA treatment significantly enhanced SOD activity in plant shoots and roots to levels higher than in the controls under saline conditions. Superoxide dismutase activity was enhanced due to JA priming under non-saline and saline conditions in plant shoot by about 77% and 209%, respectively, as compared to their corresponding untreated controls (Figure 5A). All investigated hormonal treatments failed to significantly enhance SOD activity in plant roots under non-saline and saline conditions. While JA significantly enhanced SOD activity under salinity stress by about 18% (Figure 5B).

All hormonal treatments showed significant increases in CAT activity in plant shoots and roots under saline conditions. Catalase activity increased by about 89%, 227%, and 147% in shoot after JGA, JA, and GA + JA, respectively, as compared to the untreated stressed control (Figure 5C). In plant roots, CAT activity under saline conditions enhanced by about 94%, 19%, and 77%, as compared with the stressed control after treatment with GA, JA, and GA + JA, respectively (Figure 5D).

All investigated hormonal treatments increased APX activity in shoots and roots significantly, as compared to the control, under saline conditions. In the best results recorded for JA treatment, the APX activity in shoots and roots was about 247% and 313% higher, respectively, than their corresponding controls. GA and the GA + JA mixture enhanced APX activity under saline conditions in shoots by about 151% and 105% and in root by about 182% and 146%, respectively, as compared to their corresponding controls (Figure 5E,F).

The two-way ANOVA indicated significant effects of salinity stress (*p* < 0.05) on SOD activity in plant roots only and highly significant effects (*p* < 0.001) on CAT and APX activity in both shoots and roots. Hormonal treatments showed highly significant effects (*p* < 0.001) on SOD activity in plant shoots and roots and significant effects (*p* < 0.05) on CAT activity in plant roots. The salinity × hormones interaction showed highly significant effects (*p* < 0.001) on CAT and APX activity in both plant shoots and roots (Table 1).

### 2.7. Nucleic Acids

**Figure 6:**
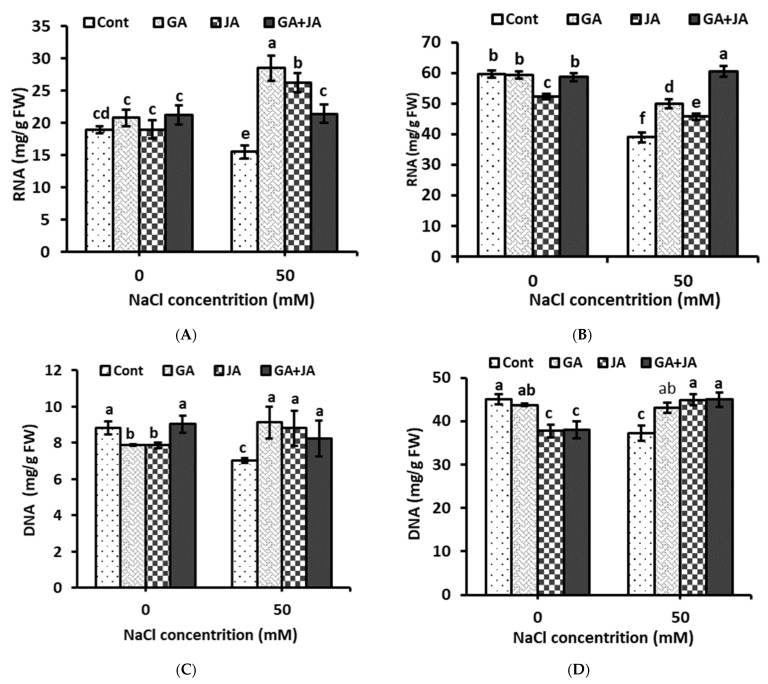
Concentration of nucleic acids—(**A**) RNA in shoot; (**B**) RNA in root; (**C**) DNA in shoot; and (**D**) DNA in root—of non-salinized and salinized summer squash plants, as affected by seed priming with 1.5 mM GA, 0.005 mM JA, and mixture of them. Data represent mean of three replicates (*n* = 3) with error bars indicating standard error of the mean. Bars carrying different lowercase letters are significantly different at *p* < 0.05 according to Duncan’s multiple range Test. *p* values for two-way ANOVA are reported in Table 1.

Based on the results demonstrated in Figure 6, salinity stress significantly reduced RNA and DNA content in summer squash shoots and roots, while all hormonal treatments significantly increased RNA concertation in plant shoots and roots to higher levels than in untreated stressed plants. In shoot samples, GA, JA, and the GA + JA mixture increased RNA concentration by about 83%, 69%, and 38%, respectively, as compared to the control (Figure 6A). In plant roots, the best treatment under saline conditions was GA + JA, followed by GA alone (Figure 6B). GA, JA, and the GA + JA mixture increased RNA concentration by about 28%, 17%, and 55%, respectively, as compared to the control. In the same context, all hormonal treatments significantly increased DNA concentration in plant shoots and roots under saline condition only (Figure 6). GA, JA, and the GA + JA mixture caused significant and similar escalations in DNA content in plant shoots (around 25%) and roots (around 20%), as compared to the stressed control (Figure 6C,D). The two-way ANOVA indicated highly significant effects (*p* < 0.001) of salinity stress and the salinity × hormones interaction on RNA and DNA concentrations in plant shoots and roots, while hormonal treatments showed significant effects (*p* < 0.05) only on DNA concentrations both in plant shoots and roots (Table 1).

## 3. Discussion

Improving the salt tolerance of crops is an essential target of plant breeders, in aiming to meet the future food demands of coming generations [23]. Pre-sowing priming with phytohormones is used to accelerate seed germination and homogenous seedling emergence, to enhance further plant growth, and to establish the stress resistance of seedlings and adult plants, which ultimately increases crop production [24]. Phytohormones interact with each other antagonistically as well as synergistically, making a super-complex network of closely intertwined pathways of biosynthesis, metabolism, transport, and signaling, thus causing responses to external stimuli [25]. Of these phytohormones, GAs and JAs have been documented as activators for plant growth under salinity stress, during which they can break seed dormancy, improve seed vitality, stimulate plant gene expression, and repair membrane damage [26,27]. The findings of this study demonstrate that salinity treatment significantly reduces the fresh and dry biomass of summer squash plants. This reduction in plant biomass under salinity stress can be attributed to reduced water absorption from the surrounding habitat as a result of physiological drought and the toxic effects of Na^+^ ions [28].

The inhibitory effect of salt stress on summer squash biomass was alleviated partially or entirely by all investigated hormonal treatments. In accordance with our results, other studies reported that treatment of plants with GA and JA is efficient in relieving salinity stress effects at various stages of plant growth by enhancing plant height, root length, root diameter, and shoot fresh weight [27,29]. Exogenous JA treatment under saline conditions may affect plant hormonal balance, e.g., ABA, which presents important evidence for understanding protection mechanisms against salt stress [30]. In the same context, the protecting influence of JA might be due to its ability to avoid the decline in cytokinin levels under salinity stress that results from the retardation of both cytokinin oxidase gene expression and activity [31]. On the other hand, GA application has been shown to improve plant growth by altering the ratio between endogenous ABA and SA, reducing the quantity of polyamines, which are involved in the regulation of aging [7].

Although Chl a content did not change significantly due to salinity stress in this study, Chl b content was significantly enhanced. Therefore, the Chl a/b ratio decreased in salinized plants. A few conflicting reports pinpointed that the amounts of Chl a and Chl b under saline conditions were more than those of the control [32]. All hormonal treatments in this study cause significant induction in Chl b and Car content, compared to the controls, under both saline and normal conditions. In accordance with our results, Misratia et al. [33] demonstrated that GA plays an important role in improving plant salt tolerance by enhancing chlorophyll biosynthesis. JA is reported also to counteract the negative effects of salinity on Chl b and Car content [34]. Nevertheless, our results showed that hormonal treatments significantly reduced Chl a content and increased Chl b content, which reduced the Chl a/b ratio. Chlorophyll b was shown to be the main constituent of the photosystems [35]. It is worth mentioning that the enhancement in Chl b content in response to hormonal treatments both under saline and non-saline conditions could be, mostly, a clear transformation of Chl a into Chl b or, at least, an improved de novo Chl b biosynthesis (Table 2). This conversion was enhanced by K fertilization under salinity stress in bitter almond trees [36]. The results of the current study also recorded the alleviating effects of GA and JA on K^+^ uptake by summer squash plants (Table 3). Several studies showed that GA and JA implement an effect on plant metabolism by regulating nutrition utilizations, mainly by meditating carbon metabolism; photosynthetic pigments were accordingly accumulated, and the content of Chl b then increased [10,12,31,34]. The transformation of Chl a into Chl b seems to be part of the overall Chl a/b interconversion cycle, which is supposed to play a major role in the formation and reorganization of the photosynthetic apparatus, and which aids plants to cope with different adverse stress conditions [37]. In their study, Yan et al. [38] proved that the decrease in the Chl a/b ratio is associated with a rise in maize crop production.

Sodium chloride stress has been shown to correlate with the disorder in Na^+^ ions homeostasis and essential minerals [39]. It has been shown that salinity stress retards plant growth owing to the excessive accumulation of Na^+^ and Cl^−^ ions in plant tissues [40]. In the current study, salt stress markedly increased Na^+^ and Cl^−^ concentrations and reduced Mg^++^ and K^+^ concentrations in summer squash shoots and roots. Related to this, Cakmak [41] demonstrated that enhancing Na^+^ content in plant leaves results in K^+^ deficiency owing to the antagonistic effects of Na^+^ and K^+^ ions. Seed priming with the studied hormonal treatments reduced Na^+^ and Cl^−^ ion accumulation to lower levels than those of the stressed control in summer squash shoots and roots. On the other hand, Mg^++^ and K^+^ concentrations in plant shoots and roots were significantly enhanced in response to seed priming with all hormonal treatments under saline conditions. In accordance with our results, one study showed that a remarkable suppression in Na^+^ and Cl^−^ accumulation was concomitant with enhancements in K^+^, Ca^++^ and Mg^++^ levels in stressed plants due to seed priming in GA3 [42]. The impact of GA3 on the mechanism of ion uptake might be allied with its influence on membrane stability, since the rate of ion uptake via the cell membrane was increased and, consequently, the translocation of ions from root to shoot was enhanced [43]. Moreover, Kang et al. [44] indicated that exogenous JA decreases Na^+^ accumulation and increases K^+^ and Mg^++^ content in salt-stressed rice plants. Balkaya et al. [45] showed that salt tolerance correlated with the ability of the plant species to accumulate higher levels of K^+^ ions. A plant’s tolerance to salinity may be more related to the K^+^/Na^+^ ratio in the cell than the absolute Na^+^ concentration [46]. In our study, all hormonal treatments significantly enhanced the K^+^/Na^+^ ratio under saline conditions to higher levels than in the stressed controls, which helps in increasing plant growth under saline conditions as shown from the results of the growth parameters in this study. More K^+^ can be taken through active transport by increasing the osmotic potential so that more water can enter the plant cell [47]. Potassium ion content in the cell is important for conservation of osmotic equilibrium, during which it activates a range of enzymes that are responsible for stomatal movement in response to changes in bulk leaf water status under salt stress [47]. Several studies have shown that plant treatments with phytohormones increase mineral uptake by enhancing root mass (as indicated from the results in Figure 1), root volume, root hair, and lateral root formation and by stimulating high levels of root activity by increasing the roots’ active absorption area [48,49,50].

Proline content in summer squash shoots and roots showed a significant increase after salinity stress. Similar observations have been reported in salt-stressed crops in several studies [51,52,53]. Proline is an important parameter to evaluate plant stress tolerance capability [54]. It is a highly water-soluble amino acid that protects cell membranes from the toxic effects of an excess of inorganic ions; in addition to its role as an osmolyte, it also helps the cells to alleviate oxidative stress in salt-affected plants [55]. Seed priming with JA and the JA + GA mixture significantly enhanced proline content in summer squash shoots, while all hormonal treatments significantly reduced proline content in root samples. The most pronounced reduction was recorded for JA treatment. Some conflicting results are available in the literature regarding the effect of JA application on proline content under water stress. Huguet-Robert et al. [56] observed that MeJA restricted proline accumulation in canola leaf discs subjected to osmotic stress. However, Maslenkova et al. [57] reported that proline was accumulated in barley plants treated with JA. In accordance with our findings, Dheeba et al. [58] detected that exogenous GA decreases the level of proline content in salt-stressed plants. The reduction in proline content after hormonal treatments indicates that these phytohormones reduce the stress caused by salinity

Thiobarbituric acid reactive substances (TBARS) are the decomposition products of the polyunsaturated fatty acids of cell membranes. Therefore, TBARS accumulation under salt stress has been used as an indicator of lipid peroxidation, which may indicate salt-induced oxidative stress [59]. In the present study, TBARS content in summer squash shoots and roots was increased markedly due to salinity stress. All hormonal treatments alleviate stress-induced lipid peroxidation in summer squash plants as inferred from the reduction in TBARS concentration in plant shoots and roots. The exogenous application of GA and JA reduced TBARS accumulation under various stress factors [60,61].

One of the most important mechanisms involved in the salt tolerance response is the harmonized up-regulation of the antioxidative system, since salt tolerance is associated with elevated activity of definite antioxidant enzymes [62]. Plants have inclusive antioxidative machinery, which plays a vital role in ROS scavenging, whereas CAT and SOD alleviate the destructive effects of oxidative stress [63]. In this study, salinity stress increased SOD activity in summer squash roots. Similar enhancement in the activity of SOD enzymes has been recorded in many plants subjected to salt stress [64]. Seed priming with JA significantly enhances SOD activity in summer squash shoots and roots under saline conditions. The increased SOD activity due to JA treatment in salt-stressed plants could be connected to its important role in plant survival. When SOD activity was elevated, the scavenging of superoxide radicals was performed properly, which protected the cell membrane against oxidative stress damage; consequently, tolerance to oxidative stress increased [65]. On the other hand, the results of this investigation showed that the activity of SOD in salinized plants significantly decreased in response to seed priming with GA. Likewise, it has been shown that the exogenous application of GA inhibits SOD activity in *Vigna radiata* plants under salt stress [43].

The down-regulation of CAT and APX activity is considered to be a general response to several stress factors [66]. Under environmental stresses, the decline in CAT activity is allegedly owing to the inhibition of enzyme synthesis or to the alteration in the assembly of enzyme subunits. In the present study, CAT and APX activity in the shoots and roots increased in hormone-treated plants to higher levels than in untreated controls under saline conditions. In support of our results, Qiu et al. [34] reported that JA significantly enhanced the CAT and APX activity under saline conditions. Overproduction of CAT and APX can be an adaptive mechanism of plants to stressful ecosystems, and JA contributes to this. The mitigation influence of the GA application on antioxidant enzyme activity has also been recorded in salt-stressed mung bean plants by Chakrabarti and Mukherji [43]. It has been noted that exogenous GA is able to overcome the influence of different salinity levels on CAT, APX, and SOD and restores their activity to values around that of the control [67].

It has been postulated that ROS, which accumulates as a result of salt stress, can damage nucleic acids [68]. The results of the current study show significant reductions in RNA and DNA contents in summer squash shoots and roots due to salinity stress. The findings of our study are in accordance with those recorded by Yupsanis et al. [69] who obtained significant decreased in RNA and DNA contents in alfalfa and lentil plants under salinity stress. In his study using five species of Chenopodiaceae, Abo-Kassem [70] concluded that the reduction in nucleic acid content, along with the enhancement in RNase activity, might be related to the increased levels of salinity which cause inhibition in the biosynthesis of nucleic acids and/or stimulation in their degradation. In the present study, seed priming with the investigated hormonal treatments increased RNA and DNA contents in shoots and roots of summer squash under saline conditions to comparable values. In support of our results, Ismail [71] recorded that salt stress reduces RNA and DNA content in sorghum plants, and pre-sowing priming of barley grains with GA enhances DNA content in salt-stressed plants. Comparable findings are recorded by Fujii et al. [72], who revealed the role of the phytohormones, especially GA, in regulating gene expression and mRNA induction by high salinity levels and the possible correlation between the endogenous GA content and the achievement of stress protection. Taken together, under salt stress conditions, the pre-sowing priming of summer squash seeds with GA or JA stabilized ionic homeostasis increased the content of chlorophyll b, carotenoid, proline, nucleic acids, and antioxidant enzyme activity and decreased the level of membrane lipid peroxidation. All these subsidized the plants and enhanced their salt tolerance. However, the GA and JA combination showed antagonistic effects for the regulation of plant growth and for tolerance responses, while JA treatment alleviated salinity stress induced in summer squash more efficiently than GA treatment or the GA + JA combination. The crosstalk between GA and JA signaling underlying this antagonistic effect needs further investigation.

## 4. Materials and Methods

### 4.1. Plant Cultivation and Hormonal Treatments

This study was conducted at King Abdulaziz University Experimental Station, Saudi Arabia during the summer season, 2018. The plants were grown in the glasshouse under natural day/night conditions with average maximum/minimum temperature of 41/25 °C and daylength of 13 h. The seeds of the summer squash (*Cucurbita pepo* L., cv. Sucheimie No. 2) were bought from Holler Co., Jeddah, Saudi Arabia. Gibberellic acid and jasmonic acid, were purchased from Sigma–Aldrich Chemie GmbH, Mittelfranken, Germany. The seeds were dispersed in plastic pots (30 cm diameter and 30 cm depth) at 1 cm depth; pot bottoms were sealed to avoid salt leaching, and each pot contained 5 kg of mixed soil, consisting of sand and pitmoss (2:1). Before sowing, summer squash seeds were primed in 1.5 mM GA, 0.005 mM JA, or a mixture of them both for 1 h at room temperature under continuous shaking. Seeds primed in distilled water were used as a control. The concentrations of GA and JA were chosen depending on a preliminary experiment, conducted using Petri dishes over 10 days with four concentrations for GA (0.1, 0.5, 1, 1.5 mM) and four for JA (0.001, 0.005, 0.01 and 0.1 mM). The best results for seed germination and seedling growth were attained when using 1.5 mM GA and 0.005 mM JA under saline conditions. In the same experiment, four concentrations of NaCl (50, 100, 150, and 200 mM) were studied and 50 mM was chosen as the effective NaCl concentration to cause moderate inhibition (around 50%) in summer squash seed germination and seedling growth (EC50 level) (see Supplementary Figures S1–S3 for more details). After pre-sowing treatments, the seeds were surface dried on filter paper and then used for cultivation. The plants were irrigated with tap water regularly every two days. Pots were arranged in a randomized complete design with three replicates. Each replicate consisted of 10 uniform plants per pot. After two weeks (3 true leaves stage), each treatment was divided into 2 sets; one of them was irrigated with 50 mM NaCl (EC: 5 dS/m) dissolved in tap water to induce salinity stress, whereas the other was irrigated with tap water. A final volume of 1500 mL of the saline solution was added to the soil, giving a final concentration of 10 mM NaCl/100 g soil. To avoid osmotic shock, 750 mL of salty solution was added at the first emergence of the third true leaf; then the other 750 mL was added 3 days later. Afterwards, irrigation was applied up to approximately 90% of the pots’ water-holding capacity, using tap water every 2 days to compensate for the loss of water due to evapotranspiration. After three weeks, plant samples were collected. For all assays, the collected shoot and root samples were frozen immediately in liquid nitrogen and kept at −80 °C.

### 4.2. Plant Biomass and Water Content

When the plants were harvested (5 weeks after planting), they were carefully separated into shoots and roots, washed using tap water and blotted with filter paper to remove excess water. Summer squash growth under different treatments was determined by measuring the fresh and dry weights in grams (g) of the shoots and roots. Dry weights were determined after drying at 70 °C to constant weight. Shoot and root water content (WC), as a percentage of fresh weight, was estimated according to Sumithra et al. [73] using the formula:

WC (%) = − [(FW − DW) × 100]/FW

### 4.3. Chlorophyll and Carotenoid Determination

Chlorophyll a, Chl b, and carotenoid were measured using UV-VIS spectroscopy according to Su et al. [74]. A total of 0.05 g of leaf tissue was suspended in 5 mL of 95% ethyl alcohol in a test tube at 60 °C, until it was colorless. Then the total volume was refilled to 5 mL with 95% ethyl alcohol. The green solution was placed in a cuvette against 1 mL of 95% ethyl alcohol as a blank. The absorbance readings were measured using spectrophotometry with a Lamda 25 UV-Vis spectrophotometer at wavelengths of 664, 649, and 470 nm.

### 4.4. Mineral Ion Determination

A total of 0.5 g of dried shoots and roots was placed in a 250 mL round bottom flask, 2 mL of concentrated nitric acid was added to the flask, and the flask was placed on a hot plate; the mixer was boiled on medium heat for 10–15 min or until the mixture was completely oxidized; afterwards, the mixture was left to cool at room temperature. A total of 1 mL of 70% perchloric acid was added to the mixture and boiled until white fumes appeared; the mixture cooled down at room temperature. A total of 5 mL of distilled water was added to the mixture, which was boiled until the white fumes stopped appearing. Finally, the solution was filtered by Whatman 1 filter paper, and the volume was refilled to 25 mL with distilled water. Then concentrations of the mineral ions were estimated via inductively coupled plasma emission optical spectrometry [75].

### 4.5. Proline Determination

Proline was measured in plant shoots and roots following the method of Bates et al. [76]. A total of 0.6 g of plant tissue was homogenized using liquid N_2_ in 1.5 mL 3% (*w*/*v*) sulfosalicylic acid and then centrifuged for 10 min at 10,000 rpm. One milliliter of the supernatant was mixed with one milliliter of ninhydrin reagent (250 mg ninhydrin, 20 mL glacial acetic acid and 30 mL of 6 M phosphoric acid) and boiled in a water bath for 1 h. The resulted color was extracted in 2 mL toluene and estimated calorimetrically at 520 nm.

### 4.6. Lipid Peroxidation Products Determination

Lipid peroxidation products were estimated by the formation of thiobarbituric acid reactive substances (TBARS) as described by Heath and Packer [77]. The crude extract was mixed with the same volume of a 0.5% (*w*/*v*) thiobarbituric acid, which contained 20% (*w*/*v*) trichloroacetic acid. Then the mixture was heated for 30 min at 95 °C, quickly cooled in an ice-bath, and centrifuged for 10 min at 3000 rpm. The absorbance of the supernatant was assayed spectrophotometrically at 532 and 600 nm TBARS concentration was calculated using the molar extinction coefficient (155 mM^−1^ cm^−1^).

### 4.7. Antioxidant Enzymes Activity Determination

Antioxidant enzymes extraction: Enzyme extraction was assayed according to Cakmak and Marschner’s method [78]. A total of 0.5 g of shoot and root tissue was ground to a fine powder in liquid (N_2_). After that, it was homogenized in 5 mL of 100 mM potassium phosphate buffer (pH 7.8), which contained 0.1 mM ethylenediamine tetraacetic acid (EDTA) and 0.1 g polyvinylpyrrolidone. The mixture was centrifuged under cooling (4 °C) for 10 min. at 18,000 rpm and the supernatants were collected and used for the analyses of enzyme activity.

Superoxide dismutase (SOD, EC 1.15.1.1) activity was assayed by following the autoxidation of epinephrine according to Misra and Fridovich [79]. Enzyme activity was assayed in a final volume of 2 mL of the reaction medium containing 25 mM of sodium carbonate buffer (pH 10.2), 200 µL 0.5 mM EDTA, and 100 µL enzyme extract. The reaction was initiated by adding 100 µL of 15 mM epinephrine (dissolved in 10 mM HCl, pH 2.4). Enzyme activity was determined by the increased absorption at 480 nm and was calculated using the molar extinction coefficient (ε = 43.6 mM^−1^ cm^−1^).

Catalase (CAT, EC 1.11.1.6) activity was assayed spectrophotometrically by monitoring the change in A240 due to the decreased absorption of H_2_O_2_ [80]. The reaction medium contained 50 mM potassium phosphate buffer (pH 7), and 500 µL of enzyme extract in a 3 mL final volume. The reaction was started by adding 100 µL of 10 mM H_2_O_2_. The enzyme activity was calculated using the extinction coefficient (ε = 39.4 mM^−1^ cm^−1^).

Ascorbate peroxidase (APX, EC 1.11.1.11) activity was determined according to Zhang and Kirkham’s method [80]. The rate of hydrogen peroxide-dependent oxidation of ascorbic acid was determined in a reaction mixture, which contained 50 mM potassium phosphate buffer (pH 7), 5 mM H_2_O_2_, 0.1 mM Na_2_-ETDA, 0.5 mM ascorbic acid, and 50 µL enzyme extract. Ascorbic acid oxidation rate was estimated from the reduction in absorbance at 290 nm. The ascorbate peroxidase activity was calculated using the molar extinction coefficient (ε = 2.8 mM^−1^ cm^−1^).

### 4.8. Nuclein Acids Determination

The determination of RNA and DNA was carried out according to the method of Schmidt and Thannhuser [81] and its modification as described by Morse and Carter [82]. A known weight of plant materials was extracted with 5% TCA, and then it was washed three times with 5 mL methanol chloroform in the ratio of 1:2; the delipidated material was dissolved in 2 mL of 1N KOH at 37 °C for 16–20 h and precipitated with 0.4 mL of 6N HCl; then it was centrifuged. The precipitate contained the DNA fraction, while the supernatant contained RNA. TCA was added to the supernatant to give the final concentration of 5% TCA. It was then centrifuged, and the supernatant constituted the RNA fraction. The precipitate was hydrolyzed in 5 mL of 5% TCA at 90 °C for 30 min, cooled, and then centrifuged, and the supernatant constituted the DNA fraction. Estimated quantitative determination of RNA and DNA, as described by Abd El-Wahab [83] and Burton [84], was carried out.

### 4.9. Statistical Analysis

All data were subjected to two-way analysis of variance (ANOVA) using the statistical software SPSS version 21.0. It was performed to examine the effects of salinity stress, hormonal treatments, and their interactions upon all investigated traits. Significant differences between mean values (*p* < 0.05) were confirmed using Duncan’s multiple range test. All values were expressed with their standard error (SE) as a mean value of three replicates.

## 5. Conclusions

This study compares the effects of exogenous GA and JA on summer squash growth and metabolic responses to figure out which phytohormone is more efficient in alleviating salinity stress in this plant. We also hypothesized that, with respect to the great abilities of GA and JA under stress, their combination would show synergistic effects in alleviating salinity drawbacks on summer squash growth by enhancing antioxidant enzymes better than GA or JA separately. The important findings of the present paper showed that GA, JA, and their mixture enhanced salt tolerance of summer squash. The first piece of evidence is that all hormonal treatments significantly enhance Chl b, carotenoid, K, and Mg contents and decreased Na and Cl contents under saline conditions. Seed priming with all hormonal treatment significantly reduced proline content in summer squash roots. All hormonal treatments alleviated stress-induced lipid peroxidation both in shoots and roots by increasing MDA content. The activity of antioxidant enzymes including SOD, CAT, and APX was enhanced due to GA and JA seed priming under non-saline and saline conditions. All investigated hormonal treatments significantly increased RNA and DNA concentration in plant shoots and roots to higher levels than in untreated stressed plants under saline conditions. Finally, the results of this research did not support our initial hypothesis: JA treatment alleviated salinity stress induced in summer squash more efficiently than GA treatment or the GA + JA combination.

## Figures and Tables

**Table 1:** The results of two-way ANOVA performed using salinity stress (0, 50 mM NaCl), hormonal treatments (GA, JA, GA + JA) and Salinity × Hormones as sources of variation.

Parameters	Significance of Sources of Variation		
	Salinity (S)	Hormones (H)	S × H
Shoot FW	***	**	***
Root FW	***	*	***
Shoot DW	***	ns	***
Root DW	***	ns	***
Shoot WC	*	ns	**
Root WC	*	ns	**
Proline in shoot	***	**	**
Proline in root	***	**	*
TBARS in shoot	***	*	*
TBARS in root	***	***	***
SOD activity in shoot	ns	***	ns
SOD activity in root	*	***	ns
CAT activity in shoot	***	ns	***
CAT activity in root	***	*	***
APX activity in shoot	***	ns	***
APX activity in root	***	ns	***
RNA in shoot	***	ns	***
RNA in root	***	ns	***
DNA in shoot	***	*	***
DNA in root	***	*	***

The stars indicate significant differences (* *p* < 0.05; ** *p* < 0.01; *** *p* < 0.001); ns, not significant.

**Table 2:** Effects of salinity stress and hormonal treatments on Chl a, Chl b, carotenoid contents, and Chl a/b ratio in summer squash shoot and root *^a^*.

NaCl (mM)	Hormones	Chl a (mg/g DW)	Chl b (mg/g DW)	Chl a/b Ratio	Carotenoid (mg/g DW)
0	Non	11.60 ± 0.08 ^a^	6.24 ± 0.11 ^f^	1.9 ± 0.03 ^b^	1.56 ± 0.07 ^f^
	GA	5.07 ± 0.52 ^ef^	30.27 ± 0.38 ^c^	0.17 ± 0.02 ^d^	1.92 ± 0.04 ^d^
	JA	5.61 ± 0.16 ^e^	39.79 ± 0.75 ^a^	0.15 ± 0.01 ^d^	2.48 ± 0.08 ^a^
	GA + JA	5.74 ± 0.52 ^e^	32.34 ± 0.88 ^c^	0.17 ± 0.02 ^d^	2.06 ± 0.05 ^c^
50	Non	11.62 ± 0.03 ^a^	16.96 ± 0.11 ^e^	0.69 ± 0.01 ^a^	1.80 ± 0.02 ^e^
	GA	7.65 ± 0.06 ^c^	29.37 ± 0.44 ^c^	0.28 ± 0.02 ^c^	2.14 ± 0.04 ^c^
	JA	9.07 ± 0.58 ^b^	35.82 ± 0.50 ^b^	0.25 ± 0.02 ^c^	2.25 ± 0.07 ^b^
	GA + JA	6.24 ± 0.39 ^d^	29.84 ± 0.92 ^cd^	0.25 ± 0.02 ^c^	2.07 ± 0.06 ^c^
ANOVA					
Salinity (S)		ns	***	**	*
Hormones (H)		***	***	***	***
S × H		***	***	***	***

*^a^* Values represent the mean ± SD, *n* = 3. Different lowercase letters indicate significant differences within parameters (*p* < 0.05) as determined by Duncan’s multiple range test. The stars indicate significant differences (* *p* < 0.05, ** *p* < 0.01, *** *p* < 0.001); ns, not significant.

**Table 3:** Effects of salinity stress and hormonal treatments on Na^+^, K^+^, Mg^++^, and Cl^−^ concentration (g/kg DW) and Na^+^/K^+^ ratio in summer squash shoot and root *^a^*.

NaCl (mM)	Hormones		Shoot						Root		
		Na^+^	K^+^	K^+^/Na^+^ Ratio	Mg^++^	Cl^−^	Na^+^	K^+^	K^+^/Na^+^ Ratio	Mg^++^	Cl^−^
0	Non	2.50 ± 0.10 ^c^	16.33 ± 0.37 ^a^	6.53 ± 0.15 ^e^	8.17 ± 0.47 ^b^	36.41 ± 2.38 ^c^	12.25 ± 0.27 ^f^	54.67 ± 7.60 ^d^	4.46 ± 0.02 ^b^	13.24 ± 2.30 ^b^	118.40 ± 10.70 ^e^
	GA	2.20 ± 0.11 _d_	11.14 ± 0.38 ^d^	5.06 ± 0.49 ^f^	8.95 ± 1.23 ^a^	35.24 ± 2.90 ^c^	12.61 ± 0.15 ^e^	44.43 ± 3.64 ^e^	3.52 ± 0.03 ^d^	15.46 ± 3.88 ^b^	115.10 ± 4.15 ^e^
	JA	1.07 ± 0.04 ^g^	11.37 ± 0.43 ^d^	10.63 ± 0.12 ^b^	7.80 ± 0.35 ^c^	25.00 ± 1.85 ^d^	10.37 ± 0.25 ^g^	73.29 ± 6.40 ^b^	7.07 ± 0.05 ^a^	10.80 ± 1.30 ^c^	143.50 ± 16.30 ^c^
	GA + JA	1.04 ± 0.01 ^g^	14.19 ± 0.27 ^c^	13.64 ± 0.14 ^a^	7.44 ± 0.53 ^c^	33.24 ± 5.57 ^c^	13.79 ± 0.56 ^e^	53.67 ± 6.20 ^d^	3.89 ± 0.01 ^c^	11.90 ± 2.57 ^c^	120.70 ± 8.32 ^d^
50	Non	3.54 ± 0.11 ^a^	13.09 ± 0.56 ^c^	3.69 ± 0.02 ^g^	6.65 ± 0.50 ^d^	54.29 ± 5.31 ^a^	54.25 ± 4.51 ^a^	44.06 ± 3.10 ^e^	0.81 ± 0.01 ^f^	11.73 ± 1.52 ^c^	251.10 ± 18.40 ^a^
	GA	2.77 ± 0.08 ^b^	15.05 ± 0.15 ^b^	5.43 ± 0.06 ^f^	6.20 ± 0.40 ^d^	44.71 ± 2.57 ^b^	23.76 ± 2.82 ^d^	59.96 ± 6.90 ^d^	2.52 ± 0.41 ^e^	13.56 ± 1.93 ^b^	198.30 ± 20.90 ^b^
	JA	1.37 ± 0.03 ^f^	11.26 ± 0.78 ^d^	8.22 ± 0.02 ^d^	7.61 ± 0.27 ^c^	55.98 ± 3.16 ^a^	32.68 ± 2.81 ^b^	83.54 ± 7.27 ^a^	2.56 ± 0.32 ^e^	21.14 ± 1.69 ^a^	185.30 ± 6.81 ^b^
	GA + JA	1.45 ± 0.01 ^e^	13.70 ± 0.10 ^c^	9.45 ± 0.12 ^c^	7.47 ± 0.67 ^c^	55.68 ± 3.54 ^a^	24.81 ± 3.27 ^c^	70.38 ± 3.37 ^c^	2.84 ± 0.31 ^e^	18.23 ± 1.98 ^a^	217.51 ± 11.63 ^b^
ANOVA											
Salinity (S)		**	**	***	**	***	***	***	***	**	***
Hormones (H)		**	**	**	ns	ns	**	**	**	ns	**
S × H		***	*	***	ns	ns	***	***	***	***	***

*^a^* Values represent the mean ± SE, *n* = 3. Different lowercase letters indicate significant differences within parameters (*p* < 0.05) as determined by Duncan’s multiple range test. The stars indicate significant differences (* *p* < 0.05, ** *p* < 0.01, *** *p* < 0.001); ns, not significant.

## Data Availability

All data are presented within the article.

## Supplementary Materials

The following are available online at https://www.mdpi.com/article/10.3390/plants10122768/s1, Figure S1. Effect of different salinity levels on seed germination and seedling growth of summer squash seedlings grown for 10 days under salt stress. All values are the mean of three replicates ± SE. Values carrying different litters are significantly different at *p* < 0.05 (A) Germination percentage (B) Plumule and radical length (C) Fresh and dry weight, Figure S2. Effect of different gibberellic acid concentrations on seedling growth of summer squash seedlings grown for 10 days under salt stress. All values are the mean of three replicates ± SE. Values carrying different litters are significantly different at *p* < 0.05 (A) Plumule length (B) Radical length (C) Fresh weight (D) Dry weigh, Figure S3. Effect of different jasmonic acid concentrations on seedling growth of summer squash seedlings grown for 10 days under salt stress. All values are the mean of three replicates ± SE. Values carrying different litters are significantly different at *p* < 0.05 (A) Plumule length (B) Radical length (C) Fresh weight (D) Dry weigh.
