# Supplementary material for: Gibberellic Acid and Jasmonic Acid Improve Salt Tolerance in Summer Squash by Modulating Some Physiological Parameters Symptomatic for Oxidative Stress and Mineral Nutrition
Source: Plants (Basel). 2021 Dec 15;10(12):2768. doi: 10.3390/plants10122768 (PMC8709387; doi:10.3390/plants10122768)

Figure S1. Effect of different salinity levels on seed germination and seedling growth of summer squash seedlings grown for 10 days under salt stress. All values are the mean of three replicates ± SE. Values carrying different litters are significantly different at *p* <0.05

(A) Germination percentage

(B) Plumule and radical length

(C) Fresh and dry weight

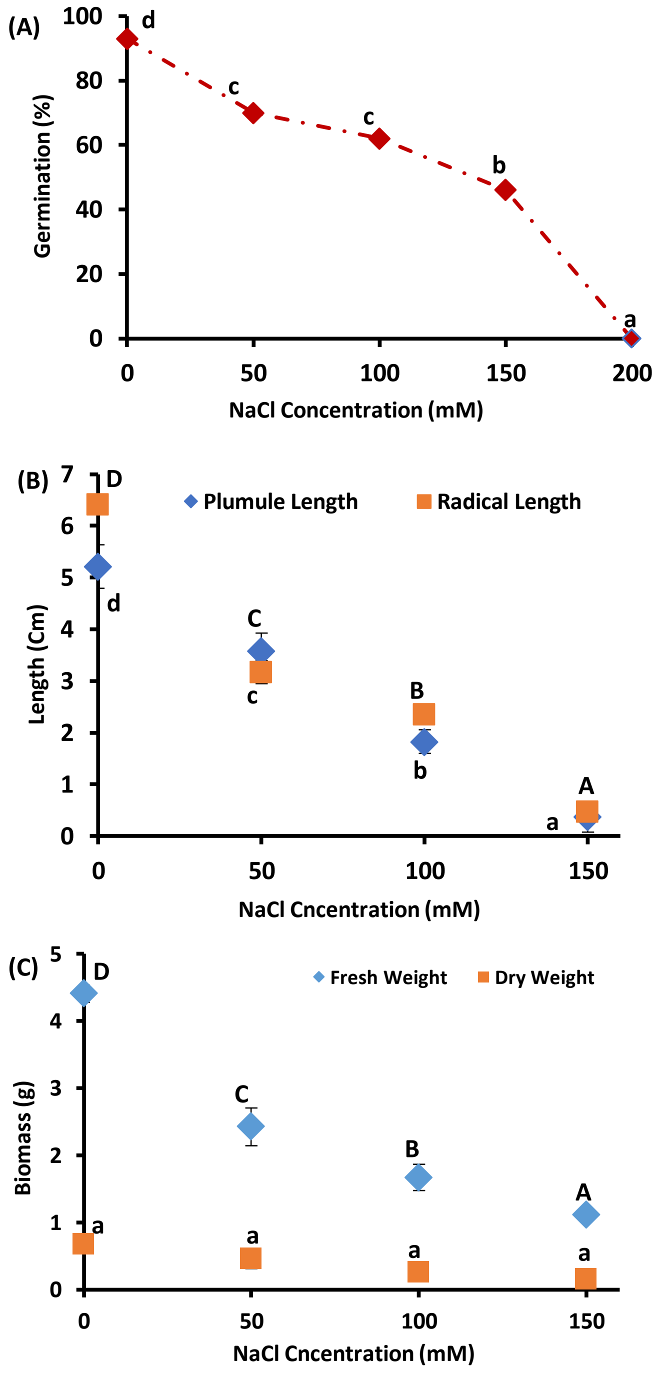

Figure S2. Effect of different gibberellic acid concentrations on seedling growth of summer squash seedlings grown for 10 days under salt stress. All values are the mean of three replicates ± SE. Values carrying different litters are significantly different at $p < 0.05$

(A) Plumule length
(B) Radical length
(C) Fresh weight
(D) Dry weigh

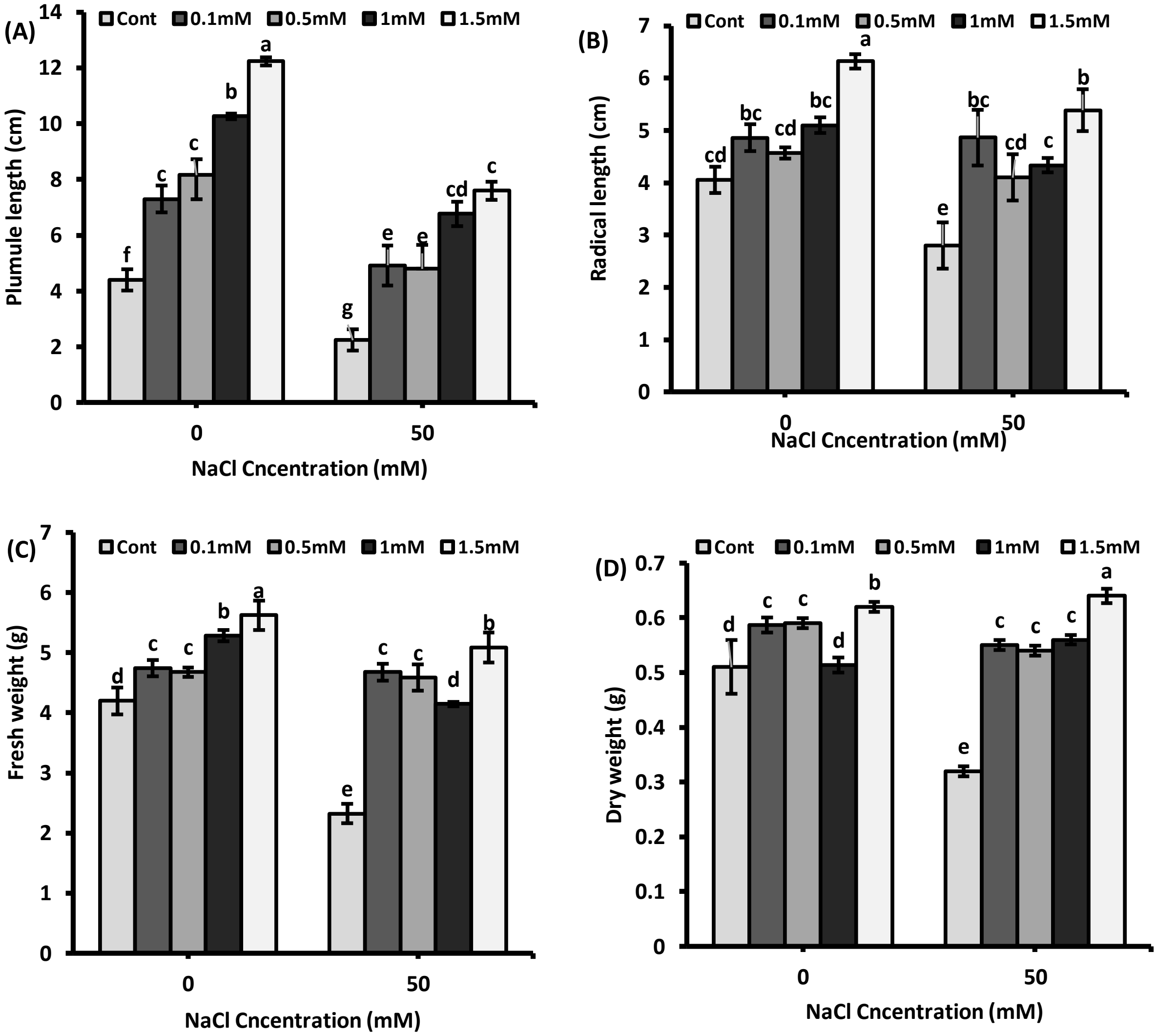

Figure S3. Effect of different jasmonic acid concentrations on seedling growth of summer squash seedlings grown for 10 days under salt stress. All values are the mean of three replicates ± SE. Values carrying different litters are significantly different at $p < 0.05$

(A) Plumule length

(B) Radical length

(C) Fresh weight

(D) Dry weigh

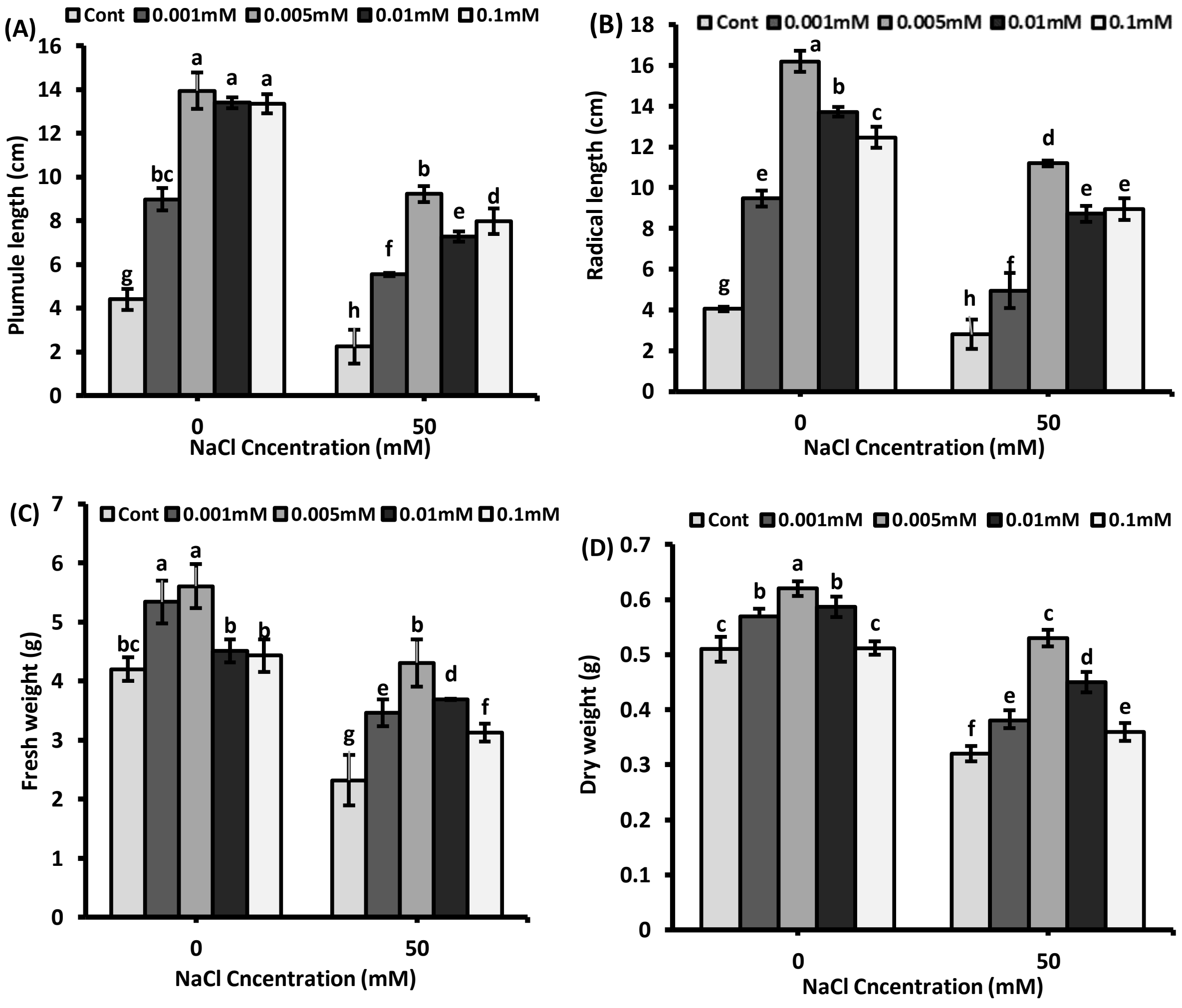

Supplement: Supplementary file 1 [file plants-10-02768-s001.zip › plants-1481096-supplementary.pdf]
